# Effective Melanoma Recognition Using Deep Convolutional Neural Network with Covariance Discriminant Loss

**DOI:** 10.3390/s20205786

**Published:** 2020-10-13

**Authors:** Lei Guo, Gang Xie, Xinying Xu, Jinchang Ren

**Affiliations:** 1 College of Information and Computer, Taiyuan University of Technology, Taiyuan 030024, China; guolei0036@link.tyut.edu.cn; 2 College of Electrical and Power Engineering, Taiyuan University of Technology, Taiyuan 030024, China; xuxinying@tyut.edu.cn; 3 Shanxi Key Laboratory of Advanced Control and Intelligent Information System, School of Electronic Information Engineering, Taiyuan University of Science and Technology, Taiyuan 030024, China; 4 Department of Electronic and Electrical Engineering, University of Strathclyde, Glasgow G1 1XW, UK

**Keywords:** melanoma recognition, embedding loss, covariance discriminant loss, deep convolutional neural network, dermoscopy image

## Abstract

Melanoma recognition is challenging due to data imbalance and high intra-class variations and large inter-class similarity. Aiming at the issues, we propose a melanoma recognition method using deep convolutional neural network with covariance discriminant loss in dermoscopy images. Deep convolutional neural network is trained under the joint supervision of cross entropy loss and covariance discriminant loss, rectifying the model outputs and the extracted features simultaneously. Specifically, we design an embedding loss, namely covariance discriminant loss, which takes the first and second distance into account simultaneously for providing more constraints. By constraining the distance between hard samples and minority class center, the deep features of melanoma and non-melanoma can be separated effectively. To mine the hard samples, we also design the corresponding algorithm. Further, we analyze the relationship between the proposed loss and other losses. On the International Symposium on Biomedical Imaging (ISBI) 2018 Skin Lesion Analysis dataset, the two schemes in the proposed method can yield a sensitivity of 0.942 and 0.917, respectively. The comprehensive results have demonstrated the efficacy of the designed embedding loss and the proposed methodology.

## 1. Introduction

Melanoma is the most-deadly type of skin cancer [1]. Fortunately, early screening of melanoma benefits the successful treatment, and the estimated 5-year survival rate is over 99% [2]. To boost the diagnostic ability, the dermoscopy technique is introduced, which can reveal the subsurface structure of the target skin region by magnifying the skin and eliminating surface reflection. Nonetheless, the manual inspection for dermoscopy images is subjective and experimental. Numerous computer-aided diagnosis methods have been presented to perform melanoma recognition [2,3,4,5,6,7,8,9,10,11,12,13,14,15,16,17,18,19].

**Figure 1:**
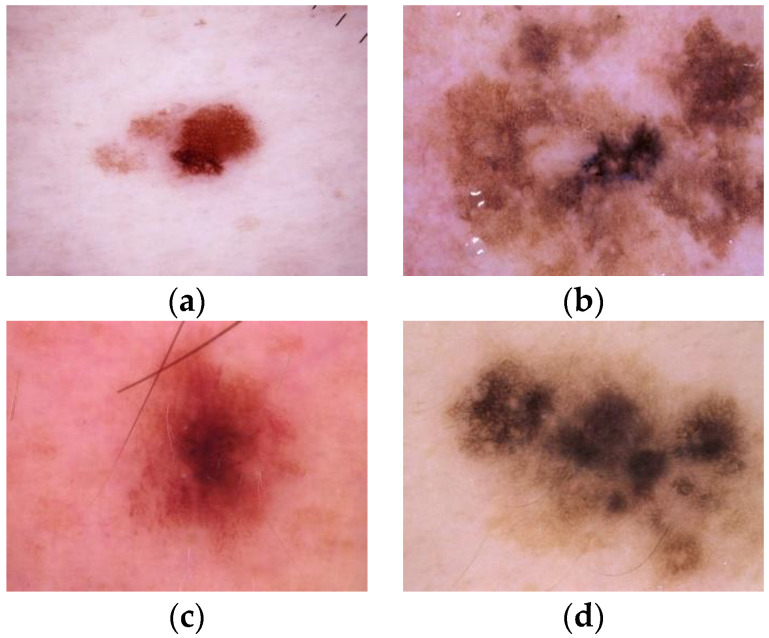
Melanoma and non-melanoma dermoscopy images. (**a**,**b**) The class of the top two images is melanoma, while (**c**,**d**) the class of the bottom images is non-melanoma. According to these four images, we can observe that there exists large intra-class variations and high inter-class similarity of the skin lesions.

Melanoma recognition is quite challenging owing to the following factors. First, the performance of melanoma recognition suffers from the data imbalance. In the current public skin lesion datasets, the number of melanoma samples is much smaller than that of the non-melanoma lesions owing to the incidence rate of melanoma. The imbalanced data distribution makes the model biased towards the non-melanoma lesions during the learning process, leading to the missing diagnosis of melanoma cases. Second, the large intra-class variations and high inter-class similarity of the melanoma and non-melanoma lesions have hindered the representation learning of the recognition model. As a fine-grained image classification task, melanoma recognition is more complicated than the general-purpose image classification problems. As seen in Figure 1, the instances from the same class actually show large variations, indicating large intra-class variations of the samples. Meanwhile, the instances from different classes such as melanoma and non-melanoma may appear quite similar, i.e., a high degree of high inter-class similarity. These have formed the second challenge in classifying melanoma and non-melanoma samples, especially for extracting discriminative features from these dermoscopy images.

The computer-aided diagnosis methods for melanoma recognition have been studied for more than twenty years [3], which regards melanoma recognition as a fine-grained image classification task. Conventional classifiers often rely on hand-crafted image features for melanoma recognition [4,5,6], which is heavily dependent on the personal experience of the researchers, yet the performance can still be limited. Recently, deep convolutional neural networks (DCNNs) have demonstrated the excellent feature representation ability in the computer vision field. Various methods [2,7,8,9,10,11,12,13,14,15,16,17,18,19] adopted DCNNs for melanoma recognition. Owing to the strong representation ability, the DCNN-based methods can generally perform the classification task effectively. In References [15,16], data imbalance is addressed with the cost-sensitive loss and Area Under the receiver operating characteristics Curve (AUC) statistics, which is directly derived from the statistic information of the input dataset. In References [20,21,22,23,24,25,26,27,28,29,30,31,32,33,34,35,36], data-level methods, algorithm-level methods, and hybrid methods were employed to address the data imbalance, boosting the performance to some extent. Hybrid methods inherit the advantages of data-level and algorithm-level methods [20]. Embedding-loss-based methods, the representative of hybrid methods, addressed the fine-grained image classification tasks in References [20,33,34,36], indicating the effectiveness in the field. Imposing embedding loss on DCNN can rectify the representation in the feature space, thus reducing the intra-class variations and inter-class similarity [34]. The embedding-loss-based methods can capture the intrinsic characteristic of the data. Inspired by the research in imbalanced classification and deep learning, a DCNN-based method with embedding loss is presented for melanoma recognition. The contributions of our work are summarized as follows:

(1) We propose a melanoma recognition approach with covariance discriminant (CovD) loss and DCNN, ensuring feature representation and classification ability for melanoma and non-melanoma. Moreover, the proposed loss has two formulations: CovD contrastive loss and CovD triplet loss.
(2) We formulate a novel embedding loss, namely covariance discriminant loss, to separate the classes in the feature space. By combing cross entropy (CE) loss and CovD loss, the recognition model can be optimized simultaneously from the views of model output and feature representation. The learned features are rectified by the CovD loss using the first and second distance, enlarging inter-class distance, and reducing the intra-class variations. Further, according to the CovD loss, we formulate the corresponding minority hard sample mining algorithm, to select the misclassified samples or samples with improper feature representation. Moreover, we analyze the relationship between CovD loss and other losses.
(3) Extensive experiments on the International Symposium on Biomedical Imaging (ISBI) 2018 Skin Lesion Analysis dataset demonstrate that the proposed loss can boost the performance consistently, and our method outperforms the comparison methods.

The remainder of the paper is organized as follows. In Section 2, the related work is summarized. In Section 3, the proposed melanoma recognition methodology with covariance discriminant loss is provided in detail. Extensive experimental results are presented in Section 4. The paper concludes in Section 5.

## 2. Related Work

### 2.1. Melanoma Recognition

The conventional computer-aided diagnosis solutions are based on the hand-crafted image features and shallow classification models. The design idea of the early features is inspired by the Asymmetry Border Color Diameter (ABCD) rule or the 7-point checklist [37]. These features usually include color features, texture features, and shape features. Rubegni et al. [4] extracted features of colors, geometries, and textures, and utilized artificial neural networks to fulfill the classification task. Similarly, Celebi et al. [5] extracted more features of color, shape, and texture features, and used support vector machines to classify the skin lesions. The extracted features in References [4,5] are global features. Local patterns are also crucial for melanoma recognition. Situ et al. [6] sliced the images into batches and employed wavelet filters to extract local features for each patch, obtaining a good classification performance. Evidently, it requires more feature engineering experiences to design these features, and the shallow models have a relatively poor classification ability for the complicated data of skin lesions.

Recently, a large number of studies based on DCNNs have shown outstanding performance for melanoma recognition [2,7,8,9,10,11,12,13,14,15,16,17,18,19]. The reason is that, compared to the shallow models, DCNN possess excellent feature representation ability. In References [7,8], DCNN was employed as the classification model, indicating the effectiveness of deep learning models for melanoma recognition. To extract more robust representations, Fisher encoding was utilized to aggregate these deep features [9]. Ge et al. [10] employed two parallel DCNNs to extract the local and global features and demonstrated the validity of the ensemble of features. In References [11,12], ensemble methods were used to fuse the predictions of different classifiers, improving accuracy. Further, the information of image segmentation was utilized in the modeling process to mitigate the negative influence of the background [13,14,15]. As such, the large intra-class variations and high inter-class similarity can be suppressed to a certain extent. In Reference [13], a two-stage method was proposed to segment the skin lesions before recognition. Yan et al. [14] used the segmentation information to regularize attention modules, focusing on the discriminative regions. In Reference [15], region average pooling was utilized to highlight relevant areas with the score map of image segmentation. However, the ground truths of image segmentation are difficult to acquire, limiting the application of these methods. Furthermore, the data imbalance has been addressed in References [15,16]. In Reference [15], a linear classifier RankOpt was used to tackle the imbalanced data distribution. In Reference [16], a weighted loss, namely the diagnosis-guided loss, was employed to strengthen the classification ability on melanoma.

### 2.2. Learning from Imbalanced Data

When the number of samples in one class is much less than that of others in a dataset, i.e., the skewed data distribution, the dataset is denoted as imbalanced [38]. The skewed data distribution may affect the recognition performance of the minority class, though the minority class is often more important in the context. This problem is crucial in melanoma recognition and other similar tasks of image classification, where a certain strategy is needed to eliminate the influence of the data imbalance. The previous related efforts can be generally categorized into data-level, algorithm-level, and hybrid methods [39]. Data-level methods are used to over-sample the minority classes or down-sample the over-represented classes [21,22]. Nonetheless, over-sampling increases the probability of overfitting, and down-sampling risks from losing useful information. Algorithm-level methods focus on weighting the training samples, including loss-based methods and cost-sensitive methods [25,26,27,28,29,30,31]. For example, Lin et al. [26] proposed focal loss to weight the hard samples. In Reference [30], Khan presented an algorithm to jointly optimize the class-dependent costs and model parameters. The computational cost of algorithm-level methods is less than that of the data-level methods. Hybrid methods integrate the data-level and algorithm-level methods to deal with the data imbalance [32,33,34,35,36]. The embedding-loss-based methods are the most commonly used hybrid methods. The contrastive loss was the first embedding loss and optimized the positive and negative distances, respectively [32]. In Reference [33], Schroff et al. performed semi-hard sampling first and employed triplet loss to optimize the relationship between positive and negative distances. In Reference [20], Huang et al. performed quintuplet sampling and employed triple-header loss to constrain the features. In Reference [34], class rectification loss was utilized to regularize the network after hard mining. The hybrid methods inherit the advantages of the other two types of methods and have a better performance.

## 3. Methodology

**Figure 2:**
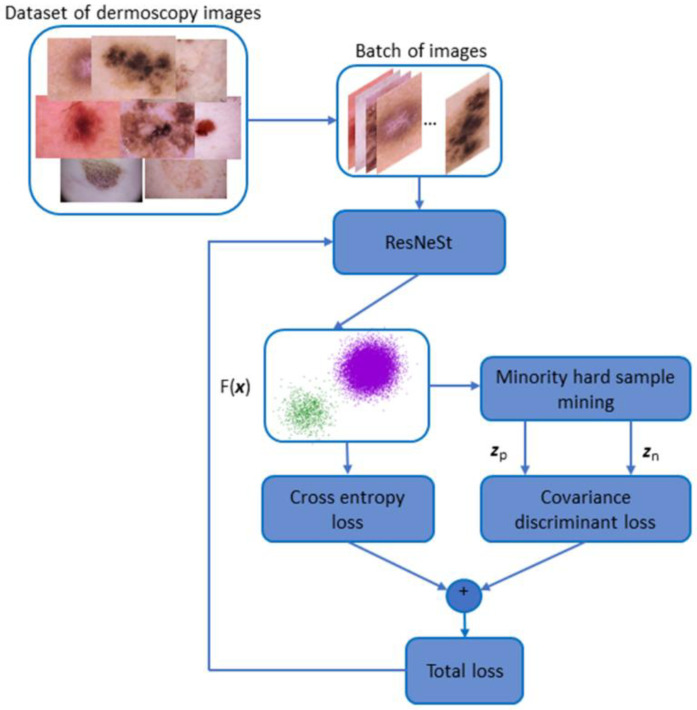
The diagram of our proposed method that combines covariance discriminant loss and cross entropy (CE) loss.

In this work, we propose a DCNN-based approach with covariance discriminant loss, as shown in Figure 2. As mentioned above, there are two challenges for melanoma recognition: data imbalance and high intra-class variations and large inter-class similarity. To circumvent the challenges, a DCNN-based method with embedding loss and CE loss is presented. Specifically, we propose an embedding loss called covariance discriminant loss to rectify the deep feature representation of hard samples. By the exploitation of the first-order and second-order distances, CovD loss produces a strong push between hard-positive samples and positive center, whist for the hard-negative samples, the loss pulls away from the minority class center. The CE loss is utilized to supervise the model output. Further, to reduce the influence of data imbalance, we use the weighted CE loss in this work. Via the supervision in these two aspects, the extracted features are more discriminant, facilitating the enhancement of recognition ability. To obtain an impressive representation ability, we leverage state-of-the-art DCNN architecture, ResNeSt [40], which incorporates split attention blocks, to extract deep features.

### 3.1. Embedding Loss

For most of the hybrid methods, the core is the embedding loss. As a result, we review the commonly used embedding losses: contrastive loss [32] and triplet loss [33]. Suppose we are given a training set $\left\{\left(z_{1},y_{1}\right),\ldots ,\left(z_{N},y_{N}\right)\right\}$, where $z_{1}\in R^{D}$, $z_{N}\in R^{D}$ are the features extracted by DCNN, and $y_{1}$, $y_{N}$ are the corresponding labels. To boost the training efficiency, we can optimize the contrastive loss on hard samples as follows:

(1) $$L_{\mathrm{Contrastive}}=y_{j}d^{2}\left(z_{a},z_{j}\right)+\left(1-y_{j}\right){\left[\alpha -d\left(z_{a},z_{j}\right)\right]}_{+}^{2}$$

where $z_{a}$ is an anchor point, $z_{j}$ is a hard sample, d( ) denotes the Euclidean distance, and α is a constant margin for hard-negative samples. If $z_{j}$ is a hard-positive sample, $y_{ij}$ = 1; otherwise, $y_{ij}$ = 0.

Compared with contrastive loss, the triplet loss considers the relationship of inter-class distance and intra-class distance. For an anchor $x_{a}$, the loss pursues that the distance between the anchor and the corresponding negative sample exceeds the distance between the anchor and the corresponding positive sample:

(2) $$L_{\mathrm{Triplet}}={\left[d^{2}\left(z_{a},z_{p}\right)-d^{2}\left(z_{a},z_{n}\right)+\alpha \right]}_{+}$$

In summary, contrastive loss and triplet loss can boost the performance of the model by imposing a constraint on the Euclidean distance of deep features.

### 3.2. Covariance Discriminant Loss

The covariance discriminant loss is designed based on contrastive loss and triplet loss. Usually, deep features are of high dimension. It is not enough to use the Euclidean distance of samples to constrain the representation of each neuron. Our starting point is that for the samples from the same class, the responses of a neuron are similar, and the co-adaptions of different neurons are also similar. Inspired by Reference [41], we use covariance to constrain the relationship of responses for different neurons. Additionally, to make the optimization objective more explicit, we compute the distance between the minority class center and hard sample, rather than the distance between the anchor and hard sample. The CovD loss has two formulations: CovD contrastive loss and CovD triplet loss. Mathematically, the CovD contrastive loss is defined as the following:

(3) $$\begin{array}{l} L_{\mathrm{CovD}\;\mathrm{Con}}=y_{ij}\frac{d^{2}\left(z_{p},\mu_{p}\right)}{M_{1}}+\left(1-y_{ij}\right){\left[\alpha -\frac{d\left(z_{n},\mu_{p}\right)}{M_{1}}\right]}_{+}^{2}\\ +\beta \cdot \left(y_{ij}\frac{{\left\|C_{p}-diag\left(C_{p}\right)\right\|}_{F}^{2}}{M_{2}}+\left(1-y_{ij}\right){\left[\alpha -\frac{{\left\|C_{n}-diag\left(C_{n}\right)\right\|}_{F}}{M_{2}}\right]}_{+}^{2}\right) \end{array}$$

(4) $$C_{p}={\left(z_{p}-\mu_{p}\right)}^{T}\left(z_{p}-\mu_{p}\right)$$

(5) $$C_{n}={\left(z_{n}-\mu_{p}\right)}^{T}\left(z_{n}-\mu_{p}\right)$$

where $z_{p}\in R^{1\times d}$ and $z_{n}\in R^{1\times d}$ are the features of hard-positive and negative samples in the current batch, $C_{p}$ and $C_{n}$ are the covariance matrixes for the hard samples, $\mu_{p}\in R^{1\times d}$ is the minority class center, β is utilized to balance the two types of terms, *M*_1_, *M*_2_ are the constants, and *diag*( ) is used to compute the corresponding diagonal matrix. For the covariance matrixes $C_{p}$ and $C_{n}$, we observe that the off-diagonal elements are to constrain the variation of the co-adaptions between different neurons, while the diagonal elements are to measure the variation of responses in a neuron. Hence, we subtract the corresponding diagonal elements. To save computation cost, the center $\mu_{p}$ is computed by averaging the features of minority class samples in the current batch. Similarly, the definition of the CovD triplet loss is:

(6) $$\begin{array}{l} L_{\mathrm{Cov}\;\mathrm{DTri}}={\left[\frac{d^{2}\left(z_{p},\mu_{p}\right)-d^{2}\left(z_{n},\mu_{p}\right)}{M_{1}}+\alpha \right]}_{+}\\ \;+\beta \cdot {\left[\frac{{\left\|C_{p}-diag\left(C_{p}\right)\right\|}_{F}^{2}-{\left\|C_{n}-diag\left(C_{n}\right)\right\|}_{F}^{2}}{M_{2}}+\alpha \right]}_{+} \end{array}$$

By minimizing the CovD loss, we can rectify the representation of the hard samples effectively, pushing the hard-positive samples towards the minority class center, and pulling the hard-negative samples away from the center. Consequently, the intra-class variations of minority class decline, and the inter-class similarity between the minority class and majority class also declines.

Stochastic gradient descent is employed to optimize the CovD loss. The gradients with respect to the parameter $\theta_{ij}$ of the DCNN for these two formulations are:

(7) $$\begin{array}{l} \frac{\partial L_{\mathrm{CovD}\;\mathrm{Con}}}{\partial \theta_{ij}^{k}}\;=2y_{ij}\left(\frac{d\left(z_{p},\mu_{p}\right)}{M_{1}}\frac{\partial d\left(z_{p},\mu_{p}\right)}{\partial \theta_{ij}^{k}}+\beta \frac{{\left\|C_{p}-diag\left(C_{p}\right)\right\|}_{F}}{M_{2}}\frac{\partial {\left\|C_{p}-diag\left(C_{p}\right)\right\|}_{F}}{\partial \theta_{ij}^{k}}\right)\\ -2\left(1-y_{ij}\right)\left(\frac{M_{1}\alpha -d\left(z_{n},\mu_{p}\right)}{M_{1}^{2}}\frac{\partial d\left(z_{n},\mu_{p}\right)}{\partial \theta_{ij}^{k}}+\beta \frac{M_{2}\alpha -{\left\|C_{n}-diag\left(C_{n}\right)\right\|}_{F}}{M_{2}^{2}}\frac{\partial {\left\|C_{n}-diag\left(C_{n}\right)\right\|}_{F}}{\partial \theta_{ij}^{k}}\right) \end{array}$$

(8) $$\begin{array}{l} \frac{\partial L_{\mathrm{CovD}\;\mathrm{Tri}}}{\partial \theta_{ij}^{k}}=2\left(\frac{d\left(z_{p},\mu_{p}\right)}{M_{1}}\frac{\partial d\left(z_{p},\mu_{p}\right)}{\partial \theta_{ij}^{k}}-\frac{d\left(z_{n},\mu_{p}\right)}{M_{1}}\frac{\partial d\left(z_{n},\mu_{p}\right)}{\partial \theta_{ij}^{k}}\right)\\ \;+2\beta \left(\frac{{\left\|C_{p}-diag\left(C_{p}\right)\right\|}_{F}}{M_{2}}\frac{\partial {\left\|C_{p}-diag\left(C_{p}\right)\right\|}_{F}}{\partial \theta_{ij}^{k}}-\frac{{\left\|C_{n}-diag\left(C_{n}\right)\right\|}_{F}}{M_{2}}\frac{\partial {\left\|C_{n}-diag\left(C_{n}\right)\right\|}_{F}}{\partial \theta_{ij}^{k}}\right) \end{array}$$

It needs to notice that the CovD loss has no impact on the parameters of the last fully connected layer for classification.

Finally, we compute the total loss by combining the CovD loss with the CE loss:

(9) $$L=L_{\mathrm{CE}}+\lambda L_{\mathrm{CovD}}$$

### 3.3. Minority Hard Sample Mining

Minority hard sample mining plays a crucial role in the CovD loss. We aim to select samples with improper representation or the misclassified samples for the minority class, which will contribute to the fast convergence of the training process. First, according to the CovD loss, we define the covariance discriminant distance:

(10) $$D\left(z,\mu_{p}\right)=\frac{d\left(z,\mu_{p}\right)}{\sqrt{M_{1}}}+\frac{{\left\|C-diag\left(C\right)\right\|}_{F}}{\sqrt{M_{2}}}$$

(11) $$C={\left(z-\mu_{p}\right)}^{T}\left(z-\mu_{p}\right)$$

As the number of positive samples (melanoma samples) is small in a batch, given the current model, we choose the misclassified samples or samples with large covariance discriminant distance from the minority class center:

(12) $${\mathrm{Pos}}_{i}=\left\{z_{i}|y_{i}=1\wedge \left({\hat{y}}_{i}\neq y_{i}\vee \;\mathrm{large}\;D\left(z_{i},\mu_{p}\right)\right)\right\}$$

Yet, the hard-negative samples, namely hard non-melanoma samples, are the misclassified samples with small covariance discriminant distance from the minority class center:

(13) $${\mathrm{Neg}}_{i}=\left\{z_{i}|y_{i}=0\wedge \left({\hat{y}}_{i}\neq y_{i}\wedge \mathrm{small}\;D\left(z_{i},\mu_{p}\right)\right)\right\}$$

In contrast to the hard-negative samples, we relax the selection condition for the hard-positive samples. At last, to keep the balance of classes, the redundant samples of hard-positive samples or hard-negative samples are discarded.

Minority hard sample mining allows the optimization to concentrate either the poor recognitions or improper feature representation, reducing the model optimization complexity. In the training process, the model is updating continuously in batch-wise, and the minority class center is updating adaptively. It is time-consuming to mine hard samples across the whole training dataset. Hence, we leverage a batch-wise way to perform hard sample mining. Moreover, hard mining is only performed for the minority class, enabling the training of the model focus on melanoma recognition. In a batch, the minority class center is calculated, and then we calculate the distances between samples and the center and perform hard mining. Finally, the proposed method is summarized in Algorithm 1.
**Algorithm 1. The Parameter Updating Algorithm****Input:** Training images X and the corresponding labels Y, parameters $\theta^{0}$ of embedding model, parameters $W^{0}$ of the classification model, balance parameter *λ*, learning rate *lr*, training epochs *L*.
**1**: *i* = 0
**2**: While *i* < *L* do: 
**3**: *i* = *i* + 1, $X_{\mathrm{Batch}},Y_{\mathrm{Batch}}=\mathrm{Sampling}\left(X,Y\right)$,
**4**: $Z_{\mathrm{Batch}}=F\left(X_{\mathrm{Batch}}\right)$,${\hat{Y}}_{\mathrm{Batch}}=C\left(Z_{\mathrm{Batch}}\right)$ //Perform feature extraction and classification.
**5**: $Z_{p},\;Z_{n}=\mathrm{Select}\left(Z_{\mathrm{Batch}},Y_{\mathrm{Batch}}\right)$ //Select positive and negative samples.
**6**: $\mu_{p}=\mathrm{Average}(Z_{p})$,$Z_{p},Z_{n}=\mathrm{Hard sample mining}(Z_{\mathrm{Batch}},Z_{p},Z_{n}),$
**7**: $L_{\mathrm{CE}}^{i}=L_{\mathrm{CE}}\left({\hat{Y}}_{\mathrm{Batch}},Y_{\mathrm{Batch}}\right)$, $L_{\mathrm{CovD}}^{i}=L_{\mathrm{CovD}}\left(Z_{p},Z_{n},\mu_{p}\right)$, $L_{\mathrm{Total}}^{i}=L_{\mathrm{CE}}^{i}+\lambda L_{\mathrm{CovD}}^{i}$,
**8**: $\theta^{i+1}=\theta^{i}-lr\cdot \frac{\partial L_{\mathrm{Total}}^{i}}{\partial \theta^{i}}$, $W^{i+1}=W^{i}-lr\cdot \frac{\partial L_{\mathrm{CE}}^{i}}{\partial W^{i}}$.//Update the model parameters.
**9**: end while
**Output**: $\theta^{L}$, $W^{L}$.

### 3.4. Relationship with Other Losses

In this subsection, we compare CovD loss with the cross-entropy loss, contrastive loss, and triplet loss. The CE loss is defined as follows:

(14) $$L_{\mathrm{CE}}=-\sum_{i=1}^{C}\sum_{j=1}^{N_{i}}y_{ij}\log{\hat{y}}_{ij}$$

where $y_{ij}$ is the label, ${\hat{y}}_{ij}$ is the prediction probability, $N_{i}$ is the sample number for class *i*, and *C* is the number of class. According to Equation (14), the CE loss is to rectify the prediction probability of the model and has less impact on the feature representation of the model.

Then, the CovD loss is compared with the contrastive loss and triplet loss. Contrastive loss is similar to the first and second terms of CovD contrastive loss, while triplet loss is similar to that of the first term of CovD triplet loss. These terms help to constrain feature representation from the perspective of the first-older distance. However, different from the contrastive loss and triplet loss, two formulations of CovD loss compute the distance between hard samples and the minority class center, possessing a more explicit optimization objective. Moreover, the corresponding residual terms of CovD loss optimize from the view of second-order information, providing another strong constraint.

## 4. Experiments

### 4.1. Dataset and Experimental Setting

Throughout this work, we conduct all the experiments on ISBI 2018 Skin Lesion Analysis dataset [42,43]. The dataset contains 10,015 dermatoscopic images, which is one of the largest publicly available skin lesion datasets. Among seven skin lesion classes contained in the dataset, i.e., melanoma, melanocytic nevus, dermatofibroma, vascular lesion, benign keratosis, basal cell carcinoma, and actinic keratosis/bowen’s disease, melanoma is selected as it is the most-deadly skin lesion. As a result, we focus on the recognition of melanoma, and all other non-melanoma lesions are regarded as another class. As there are 1113 melanoma samples in the dataset, the number of non-melanoma lesions becomes 8902, i.e., a severe imbalance of the data distribution between the two classes. The resolution of the images is 600 × 450. Pathological verification is performed on 53.3% of the images, and 1/2 of the images for a class are used for training, 1/4 of images are utilized for validation, and the remaining images are employed for testing.

The experiments are implemented on a workstation equipped with 2 NVIDIA Titan Xp GPUs.

### 4.2. Evaluation Metrics

Five metrics are adopted for quantitative performance evaluation, including the sensitivity, specificity, accuracy, Receiver Operating Characteristics (ROC) curve, and Area Under the ROC Curve (AUC). The definitions of the sensitivity, specificity, and accuracy are given as follows:

(15) $$sensitivity=\frac{tp}{tp+fn}$$

(16) $$specificity=\frac{tn}{fp+tn}$$

(17) $$accuracy=\frac{tp+tn}{tp+fp+tn+fn}$$

where *tp*, *tn*, *fp*, and *fn* are the numbers of true positives, true negatives, false positives, and false negatives, respectively [42].

In addition, ROC curve is a plot of the paired values between sensitivity and 1-specificity at various parameters. AUC, another global metric, denotes the area under the ROC curve. Sensitivity is the true positive rate, indicating the ability to correctly classify samples with melanoma. Specificity means the true negative rate, indicating the ability to correctly recognize non-melanoma samples. Accuracy is a global indicator, measuring the ability to correctly identify samples in the two classes. As another two types of global indicators, the ROC curve and AUC measures the overall recognition performance of the designed algorithm. As the purpose of the work is to recognize melanoma, sensitivity is more clinically significant, compared to other metrics.

### 4.3. Ablation Study

In Table 1, the effectiveness of the CovD loss is explored first. We choose the method with CE loss as the baseline, and we also compare with the method using CE and contrastive loss, and the method using CE and triplet loss. First, we observe that by combining CE loss with embedding loss, the performance can be improved in terms of the specificity, accuracy, and AUC. However, the sensitivity decreases in the last two methods. Second, after introducing the new constraint, the proposed combined loss outperforms the one with only the embedding loss in terms of sensitivity and AUC. The ablation study has suggested that the proposed CovD loss can improve the performance of melanoma recognition.

**Figure 3:**
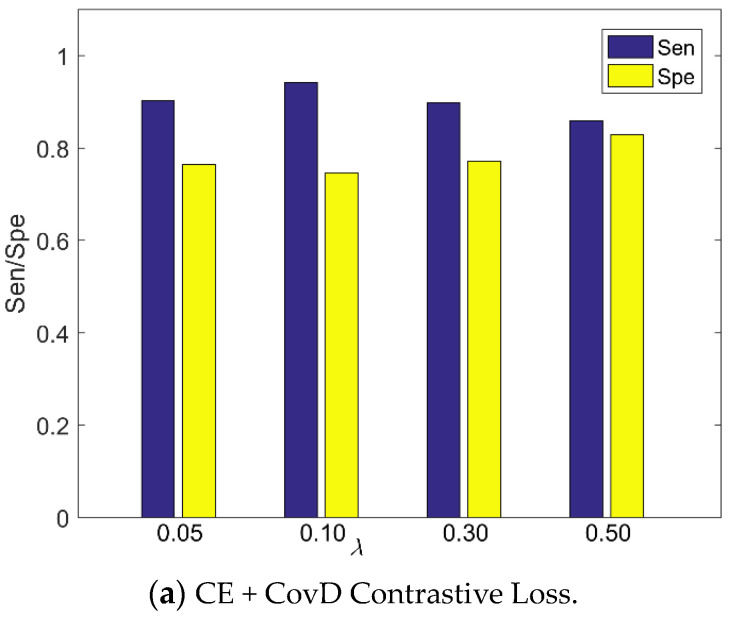

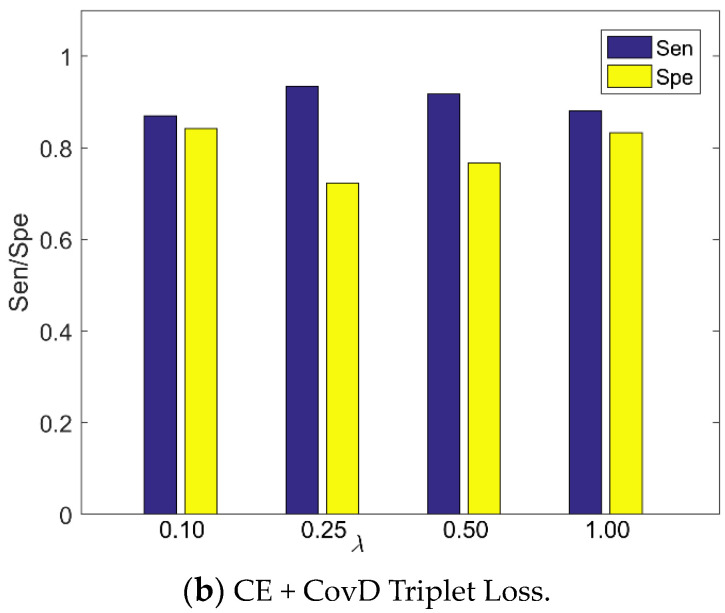
The effect of λ on our method using CE + CovD loss.

We investigated the effect of λ for the proposed method based on CE and CovD Loss, shown in Figure 3. The hyperparameter λ balances the CE loss and the embedding loss. According to Figure 3a, when λ ranges in [0.05, 0.50], our presented method with CE and CovD contrastive loss has a good performance, demonstrating its robustness. According to Figure 3b, we can have similar results for the proposed method based on CovD Triplet Loss.

### 4.4. Comparison with Other Methods

To further verify our method, our method is compared with four other methods, including hybrid deep neural networks (HDNN) [12], the method using global and local features [10], ensemble method [11], and the patch-based attention method [16]. HDNN [12] augmented the training set first, employed three pre-trained models to extract features, fed the features to the Support Vector Machines (SVMs), and finally fused the outputs. The method using global and local features [10] is based on the outer product of features and deep features. The ensemble method [11] used the ensemble of four deep learning models to improve sensitivity. The patch-based attention method [16] leveraged a weighted loss to deal with the data imbalance.

**Figure 4:**
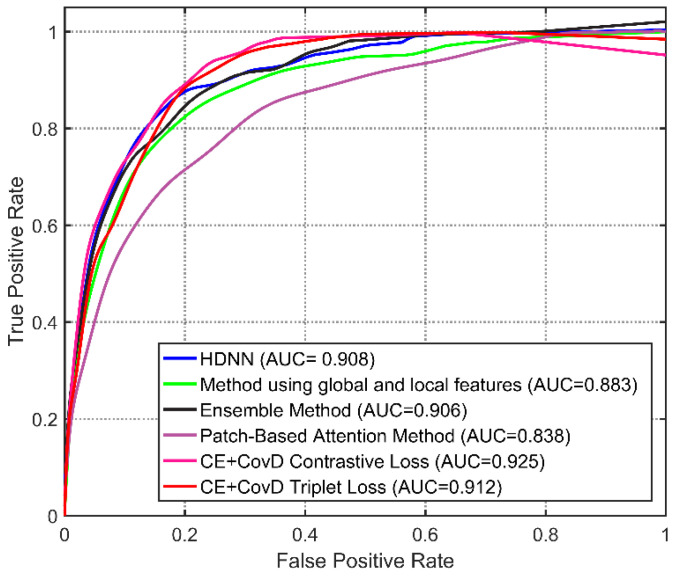
ROC curves and AUC of our method and the other methods.

The experimental comparisons are given in Table 2 and Figure 4. First, HDNN performs not very well. The reason is that the classifiers in HDNN are based on the features extracted by the pre-trained model, which is not discriminant enough. Our method performs better on the ROC curve, sensitivity, and AUC. The ROC curves of our method form an upper envelope over those of the other methods, indicating the superiority. Our method with CovD loss exceeds the comparison methods slightly on AUC. Further, the sensitivity of our method is higher by at least 0.029 than that of the comparison methods. Overall, sensitivity is the core metric, and a high sensitivity means less missed melanoma detection. Therefore, we draw the conclusion that our method outperforms the comparison methods.

We also compare the computational cost, as shown in Table 3. In contrast to the methods with multiple models, our method and the patch-based attention method with a single model need significantly less time to train on the same computational hardware. Though the feature extraction of HDNN is based on the pre-trained models, HDNN requires more time to train the SVMs with the augmented dataset.

### 4.5. Feature Visualization

**Figure 5:**
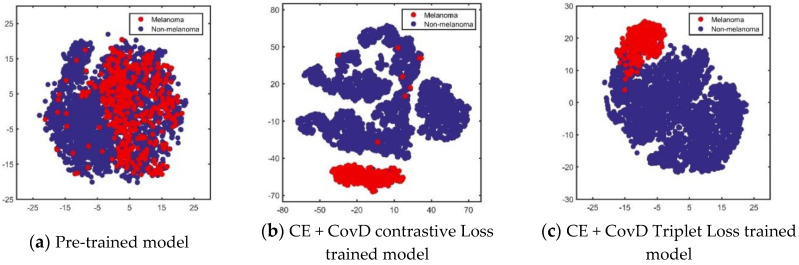
Feature visualization by t-Distributed Stochastic Neighbor Embedding (t-SNE).

In Figure 5, we visualize the features extracted from the pre-trained model, CE + CovD contrastive loss trained model, and the CE + CovD triplet loss trained model. As can be seen, for the pre-trained model, the features of melanoma and non-melanoma mix together, which is difficult for classification. With our proposed methodology, the features of melanoma show reduced intra-class variations and low similarity to the features of non-melanoma. To this end, we can conclude that the proposed method is effective for refining feature extraction for the classification of melanoma.

### 4.6. Discussion

For melanoma recognition, the data imbalance and improper intra-class variations and inter-class similarity are the main issues. In this work, we employed a CovD-loss-based method to address the two issues simultaneously. The CovD loss is based on the informative hard samples, reducing the model optimization complexity. According to the experimental result, sensitivity and AUC are high, but the accuracy and specificity are relatively low. The proposed method misclassifies a fair number of non-melanoma instances into melanoma. However, the kind of misclassification is acceptable clinically. The training computational cost is a key factor for deep learning solutions. Compared with the methods in References [10,11], we employed a single end-to-end model, avoiding the complicated ensemble process of features or models.

## 5. Conclusions

In this paper, we proposed a DCNN-based method with the covariance discriminant loss for melanoma recognition, which jointly employs cross-entropy loss and covariance discriminant loss to constrain the training from the views of model output and feature representation. Concretely, we designed a novel embedding loss called covariance discriminant loss, which can provide additional constraints compared with the contrastive loss and triplet loss. We also formulated the corresponding minority hard sample mining algorithm. We conducted all the experiments on the ISBI 2018 Skin Lesion Analysis dataset. The experimental results demonstrated that the presented method possesses excellent performance for melanoma recognition.

Further work includes integrating Bayesian theory, weakly supervised image segmentation, and other factors into our melanoma recognition method. Weakly supervised image segmentation relies on the image-level ground truths, and can boost the recognition performance [44,45]. In addition, quantifying the uncertainty is another key factor for the computer-aided diagnosis methods. Based on the Bayesian theory, we can estimate the uncertainty of the predicted results [46,47]. The proposed approach is actually a data-driven method. When applying it in the clinic, the difference of distribution between the real local data and the training data needs be considered. If the difference is too large, the model needs be adapted and even re-trained using the new data from scratch [48].

## Figures and Tables

**Table 1:** The effect of different embedding losses.

Method	Sensitivity	Specificity	Accuracy	AUC
CE	0.914	0.536	0.578	0.822
CE + Contrastive Loss	0.831	0.838	0.837	0.922
CE + Triplet Loss	0.874	0.752	0.765	0.896
CE + CovD Contrastive Loss	0.942	0.747	0.769	0.925
CE + CovD Triplet Loss	0.917	0.767	0.784	0.912

**Table 2:** Experimental result of our method and the other methods.

Method	Sensitivity	Specificity	Accuracy	AUC
HDNN [12]	0.417	0.972	0.911	0.908
Method using global and local features [10]	0.745	0.863	0.850	0.883
Ensemble Method [11]	0.759	0.869	0.857	0.906
Patch-Based Attention Method [16]	0.888	0.562	0.625	0.838
CE + CovD Contrastive Loss	0.942	0.747	0.769	0.925
CE + CovD Triplet Loss	0.917	0.767	0.784	0.912

**Table 3:** Training time comparison of our method and the other methods.

**Method**	**Training Time/hour**
HDNN [12]	4.63
Method using global and local features [10]	3.32
Ensemble Method [11]	3.23
Patch-Based Attention Method [16]	1.91
CE + CovD Contrastive Loss	1.58
CE + CovD Triplet Loss	1.63
